# Author Correction: A consensus *S. cerevisiae* metabolic model Yeast8 and its ecosystem for comprehensively probing cellular metabolism

**DOI:** 10.1038/s41467-020-19358-9

**Published:** 2020-10-22

**Authors:** Hongzhong Lu, Feiran Li, Benjamín J. Sánchez, Zhengming Zhu, Gang Li, Iván Domenzain, Simonas Marcišauskas, Petre Mihail Anton, Dimitra Lappa, Christian Lieven, Moritz Emanuel Beber, Nikolaus Sonnenschein, Eduard J. Kerkhoven, Jens Nielsen

**Affiliations:** 1 Department of Biology and Biological Engineering, Chalmers University of Technology, Kemivägen 10, SE412 96 Gothenburg, Sweden; 2 School of Biotechnology, Jiangnan University, 1800 Lihu Road, 214122 Wuxi, Jiangsu China; 3 The Novo Nordisk Foundation Center for Biosustainability, Technical University of Denmark, DK-2800 Kgs Lyngby, Denmark; 4 BioInnovation Institute, Ole Maaløes Vej 3, DK2200 Copenhagen N, Denmark

**Keywords:** Genomic analysis, Biochemical networks, Computational models

Correction to: *Nature Communications* 10.1038/s41467-019-11581-3, published online 8 August 2019.

In the original version of this Article, the calculation of the WAP score in the second paragraph of the “Methods” subsection “CLUMPS method to calculate *p* values of mutation-enriched PDB files” was incorrectly written as “Next, assuming that the uniform distribution of mutations across the protein residues covers the given structure, calculate each WAP score in 10 randomisations to obtain the null distribution.”  It should read “Next, assuming that the uniform distribution of mutations across the protein residues covers the given structure, calculate each WAP score in 10^4^ randomisations to obtain the null distribution.”

This has been corrected in both the PDF and HTML versions of this Article.

