# Supplementary material for: A consensus S. cerevisiae metabolic model Yeast8 and its ecosystem for comprehensively probing cellular metabolism
Source: Nat Commun. 2019 Aug 8;10:3586. doi: 10.1038/s41467-019-11581-3 (PMC6687777; doi:10.1038/s41467-019-11581-3)
Supplement: Supplementary file 1 — Supplementary Information [file 41467_2019_11581_MOESM1_ESM.pdf]

A consensus S. cerevisiae metabolic model Yeast8 and its ecosystem for comprehensively probing cellular metabolism

Hongzhong Lu[1]*, Feiran Li[1]*, Benjamín J Sánchez[1], Zhengming Zhu[1,2], Gang Li[1], Iván Domenzain1, Simonas Marcišauskas[1], Petre Mihail Anton[1], Dimitra Lappa[1], Christian Lieven[3], Moritz Emanuel Beber[3], and Nikolaus Sonnenschein[3], Eduard J Kerkhoven[1], Jens Nielsen[1,3,4#]

[1] Department of Biology and Biological Engineering, Chalmers University of Technology, Kemivägen 10, SE412 96 Gothenburg, Sweden

[2] School of Biotechnology, Jiangnan University, 1800 Lihu Road, Wuxi, Jiangsu 214122, China [3] The Novo Nordisk Foundation Center for Biosustainability, Technical University of Denmark, DK-2800 Kgs. Lyngby, Denmark

[4] BioInnovation Institute, Ole Maaløes Vej 3, DK2200 Copenhagen N, Denmark

[*] These authors contributed equally to this work.
[#] Correspondence to: nielsenj@chalmers.se

Table of Contents

Supplementary Figure ***2***

Supplementary Tables ***13***

Supplementary Methods ***20***

- **Quality improvements for Yeast7.6** **20**
- **Adding new GPRs by merging Yeast7.6, iTO977 and iSce926** **21**
- **Adding new GPRs based on gene function annotation in 5 databases** **21**
- **Improve the gene coverage for transport reactions based on gene annotation in TCDB** **22**
- **Adding new reactions based on Biolog experiments** **23**
- **Adding new reactions based on metabolomics mapping** **24**
- **Adding new reactions based on gap filling** **24**
- **Improving the biomass equation** **24**
- **Comparison with historical yeast models** **25**
- **Protein existence analysis based on proteomics data** **25**

Supplementary Note 1 .... ***26***

- **Pan-genome re-annotation** **26**
- ***In silico* growth simulation of coreYeast8** **26**
- ***In silico* maximal yield analysis of 20 amino acids using the simulation of 1011 strain specific models** **26**

Supplementary Note 2 .... ***27***

- **Refining the quality of protein 3D structures** **27**

Supplementary References ***28***

Supplementary Figure

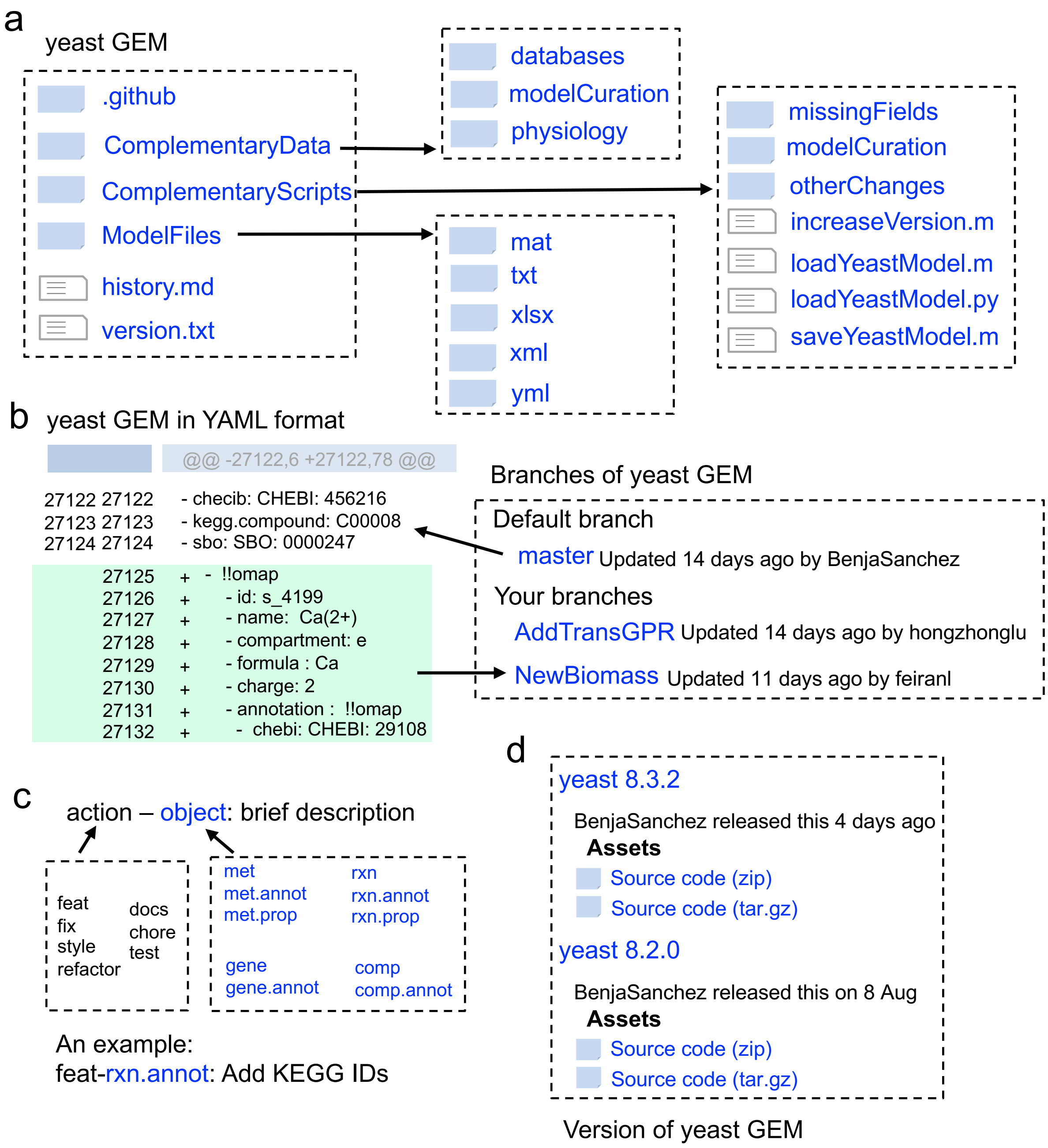

Supplementary Figure 1 Repository of yeast GEM on GitHub. (a) Main contents of the yeast GEM project. (b) Everything is recorded using Git. (c) Commit format to record changes. (d) Versions of yeast GEM on GitHub were recorded.

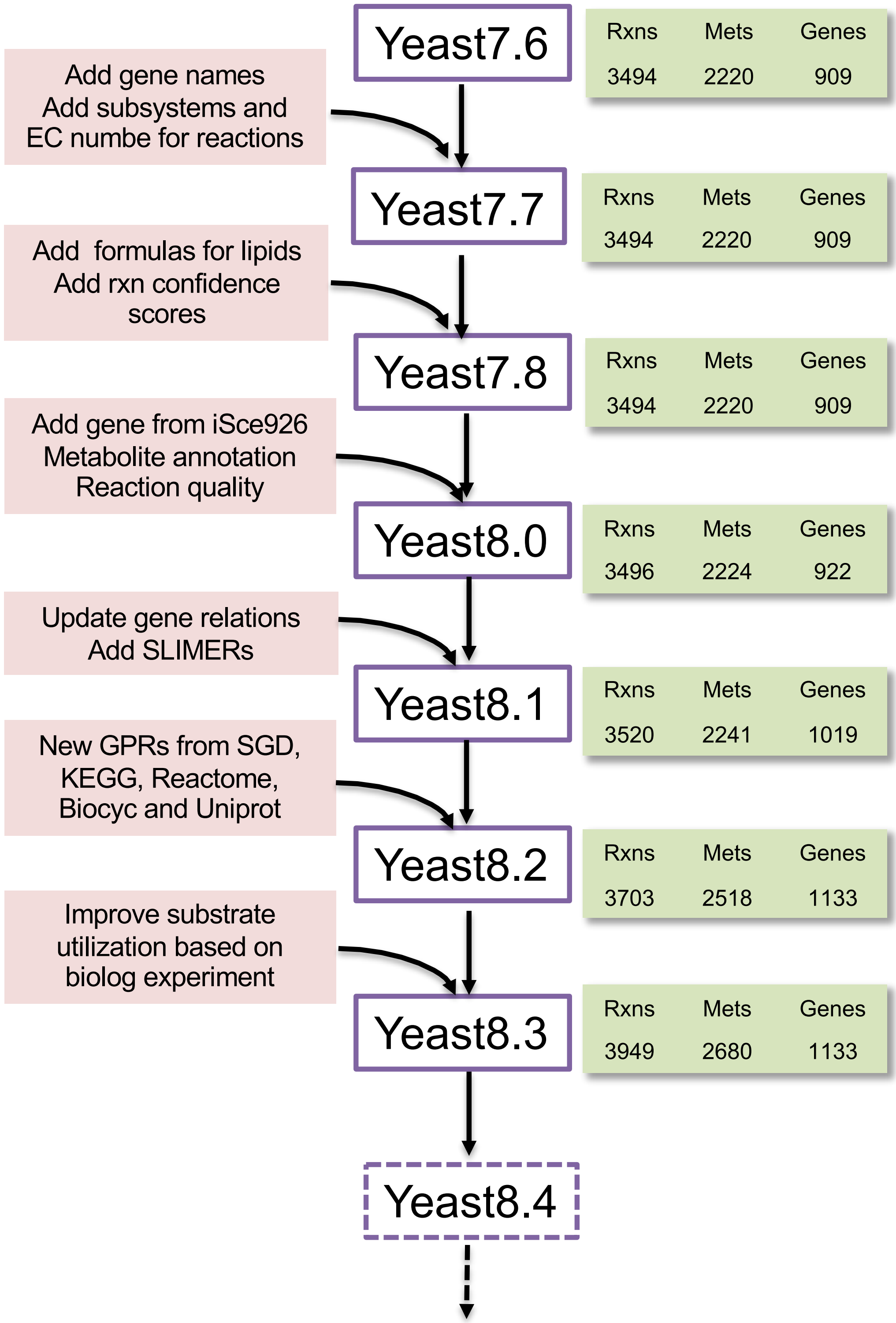

Supplementary Figure 2 Detailed procedures to improve Yeast7.6 to Yeast8. Additional details for each update can be found in https://github.com/SysBioChalmers/yeast-GEM/releases.

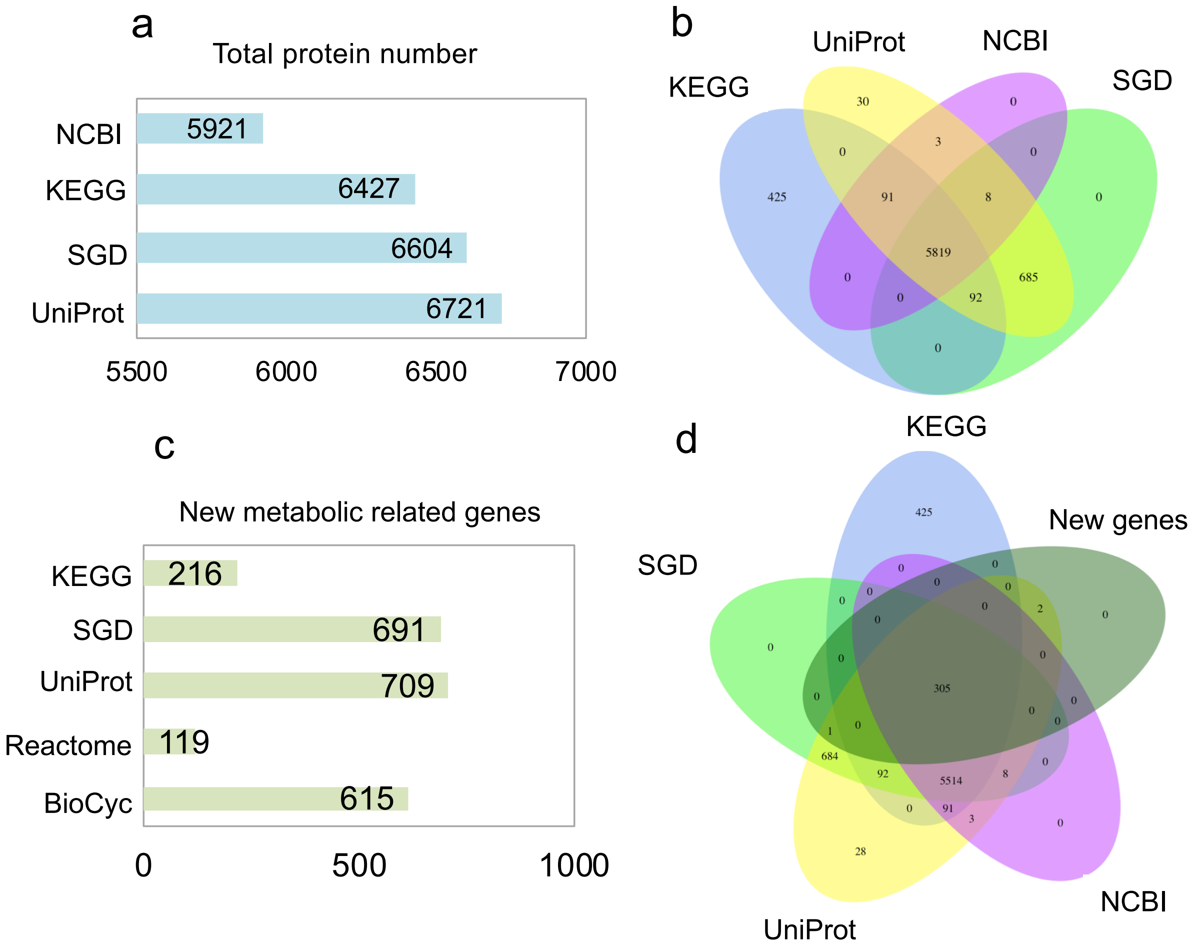

Supplementary Figure 3 Adding GPRs based on gene annotation in several databases related to *S. cerevisiae* S288c. (a) Summary of protein number from four databases. (b) Venn graph for the gene annotation from KEGG, UniProt, NCBI and SGD database. (c) Metabolic related genes which were not present in Yeast7. (d) Analysis of new genes through comparison with four main databases.

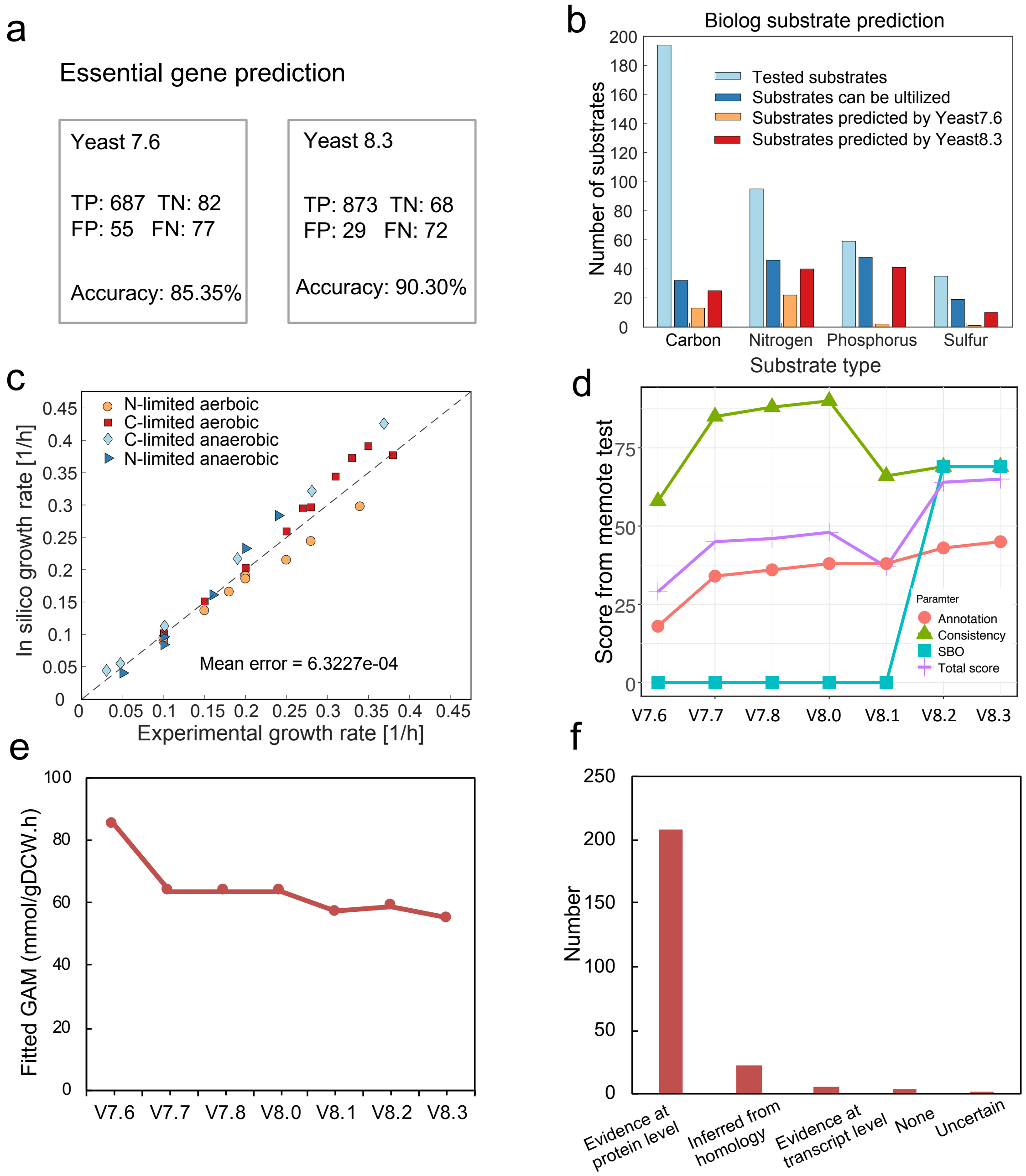

Supplementary Figure 4 Model validation of Yeast8. (a) Gene essentiality analysis. (b) Substrate usage analysis. (c) Growth predictions in chemostat conditions. (d) Test scores of yeast GEM based on the Memote test suite (the decrease of score in Yeast8.1 is due to the addition of SLIME reactions). (e) Fitted growth associated maintenance (GAM) for different versions of yeast GEM. (f) Gene annotation evidences from the UniProt database.

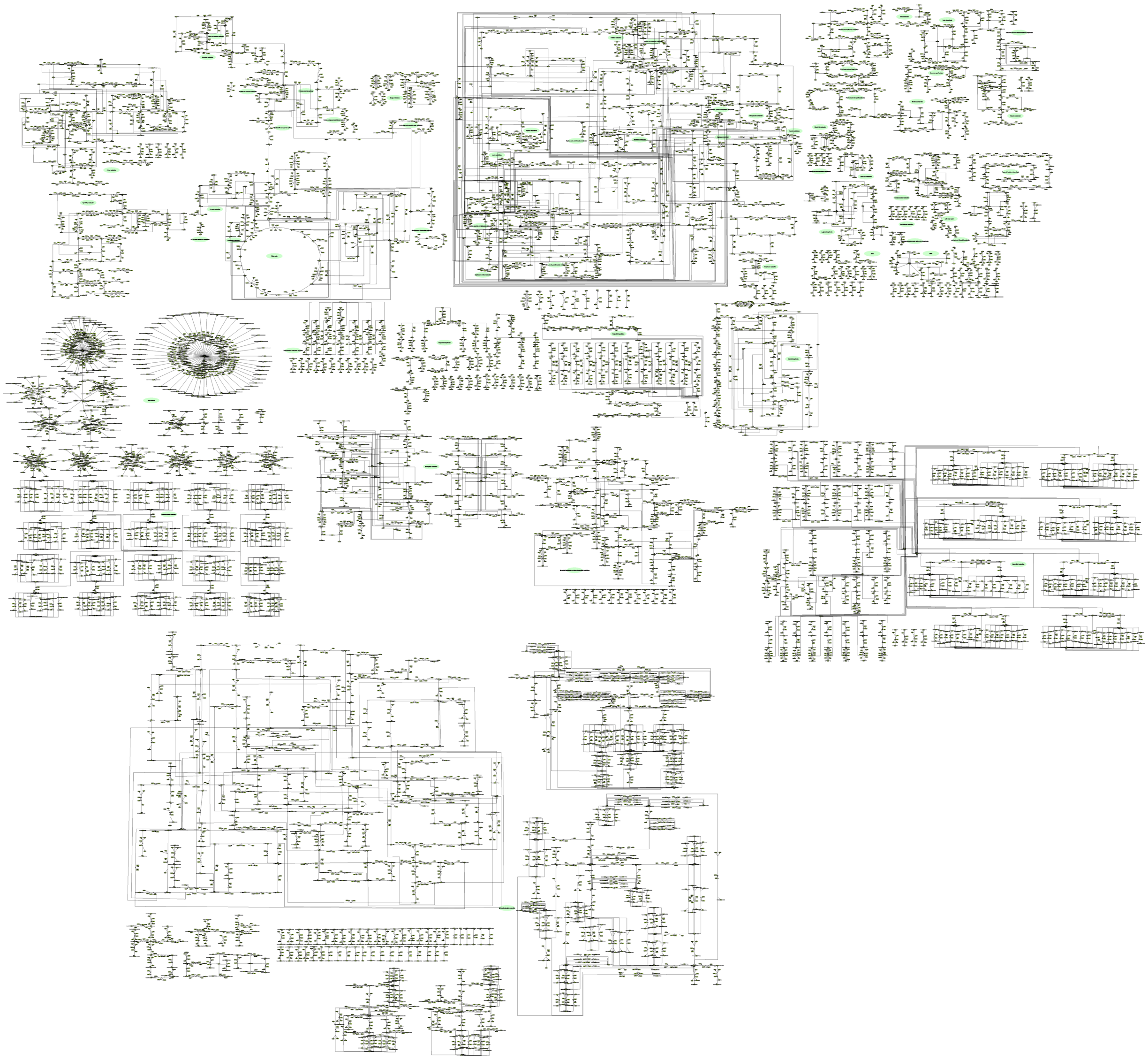

Supplementary Figure 5 Map of Yeast8 drawn by CellDesigner 4.4. It can be also found in https://github.com/SysBioChalmers/Yeast-maps/tree/master/SBMLfiles

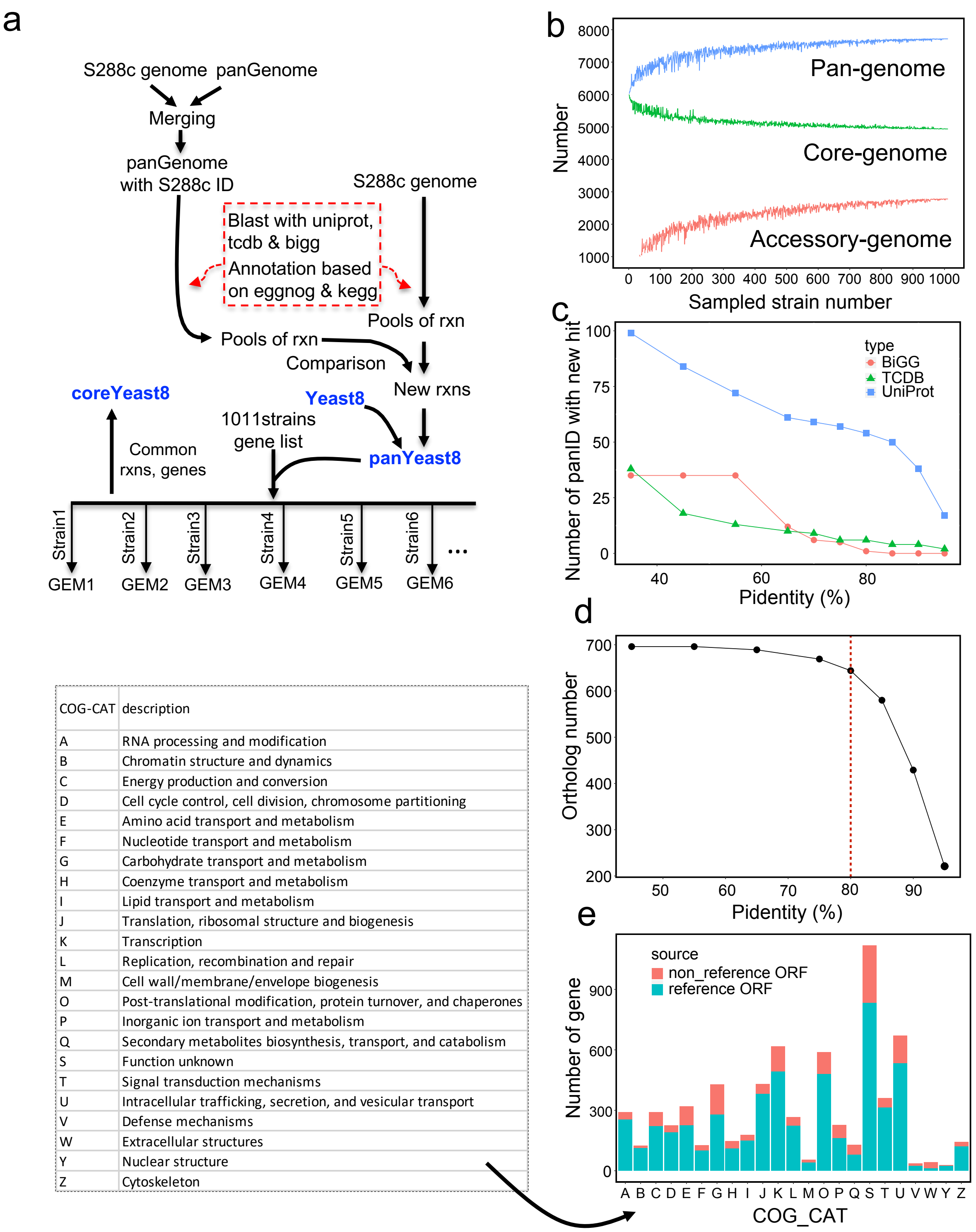

COG-CAT	description
A	RNA processing and modification
B	Chromatin structure and dynamics
C	Energy production and conversion
D	Cell cycle control, cell division, chromosome partitioning
E	Amino acid transport and metabolism
F	Nucleotide transport and metabolism
G	Carbohydrate transport and metabolism
H	Coenzyme transport and metabolism
I	Lipid transport and metabolism
J	Translation, ribosomal structure and biogenesis
K	Transcription
L	Replication, recombination and repair
M	Cell wall/membrane/envelope biogenesis
O	Post-translational modification, protein turnover, and chaperones
P	Inorganic ion transport and metabolism
Q	Secondary metabolites biosynthesis, transport, and catabolism
S	Function unknown
T	Signal transduction mechanisms
U	Intracellular trafficking, secretion, and vesicular transport
V	Defense mechanisms
W	Extracellular structures
Y	Nuclear structure
Z	Cytoskeleton

Supplementary Figure 6 panGenome annotation to generate panYeast8 and 1011 ssGEMs. (a) Pipeline to reconstruct panYeast8 and 1011 ssGEMs. (b) Number of core genes, accessory genes and pan-genes when the number of sampled strains increased. (c) New hits from pan-genome in blast analysis which were not present in the reference genome of *S. cerevisiae S288c* strain. (d) Ortholog gene relation check based on the BBH analysis. (e) panGenome annotation based on EggNOG web service.

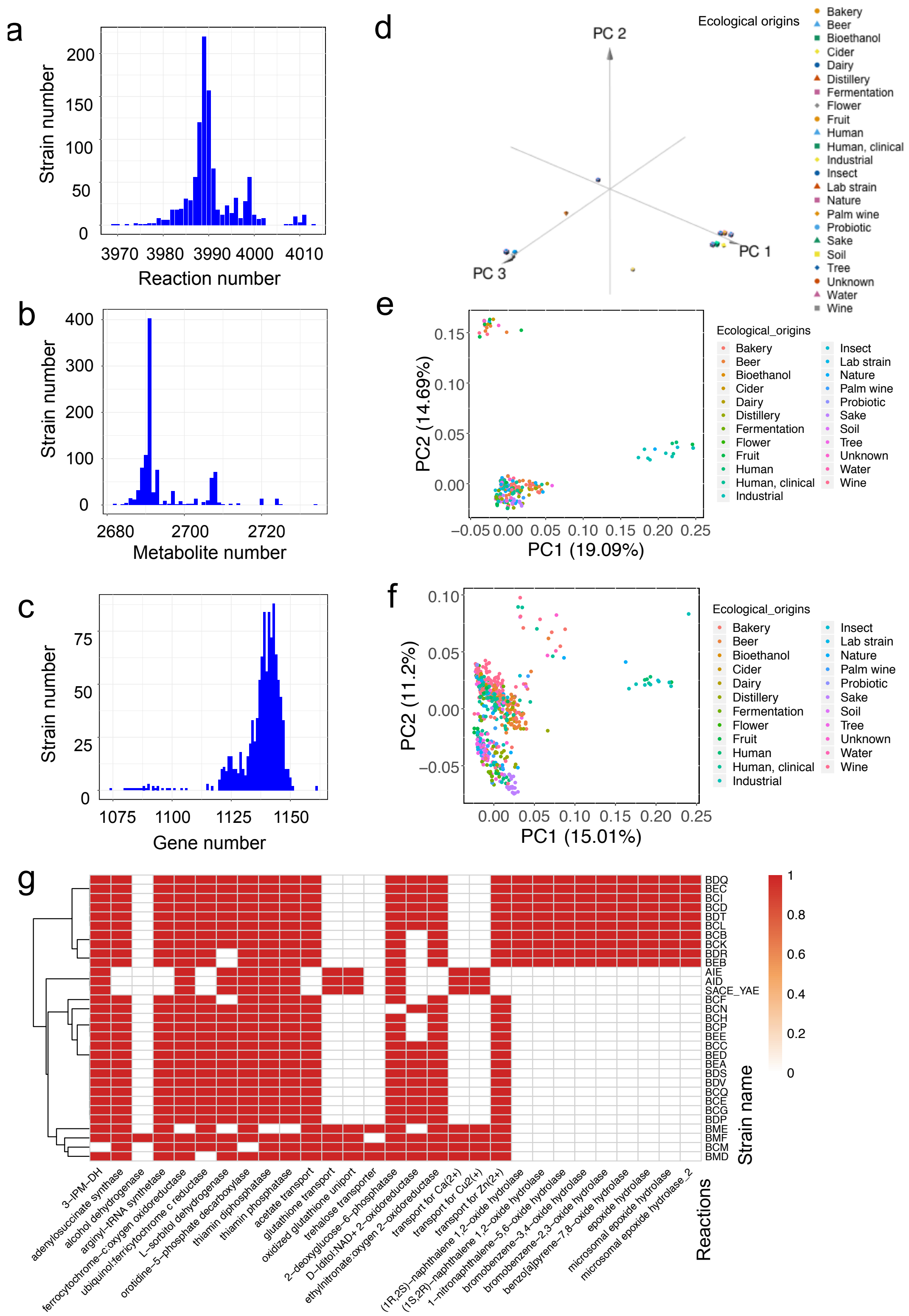

Supplementary Figure 7 Strain classification analysis using gene and reaction existence matrix from 1,011 ssGEMs and the model simulation. (a) Reaction number distribution in the 1,011 ssGEMs. (b) Metabolite number distribution in the 1,011 ssGEMs. (c) Gene number distribution in the 1,011 ssGEMs. (d) 3D PCA analysis based on the yields of 26 metabolites on glucose. (e) PCA analysis of all strains based on reactions existence in the 1,011 ssGEMs. (f) PCA analysis based on genes existence in the 1,011 ssGEMs. (g) Classification of yeast strains from ecological origin ”Human” based on the reactions existence in ssGEMs.

a

b

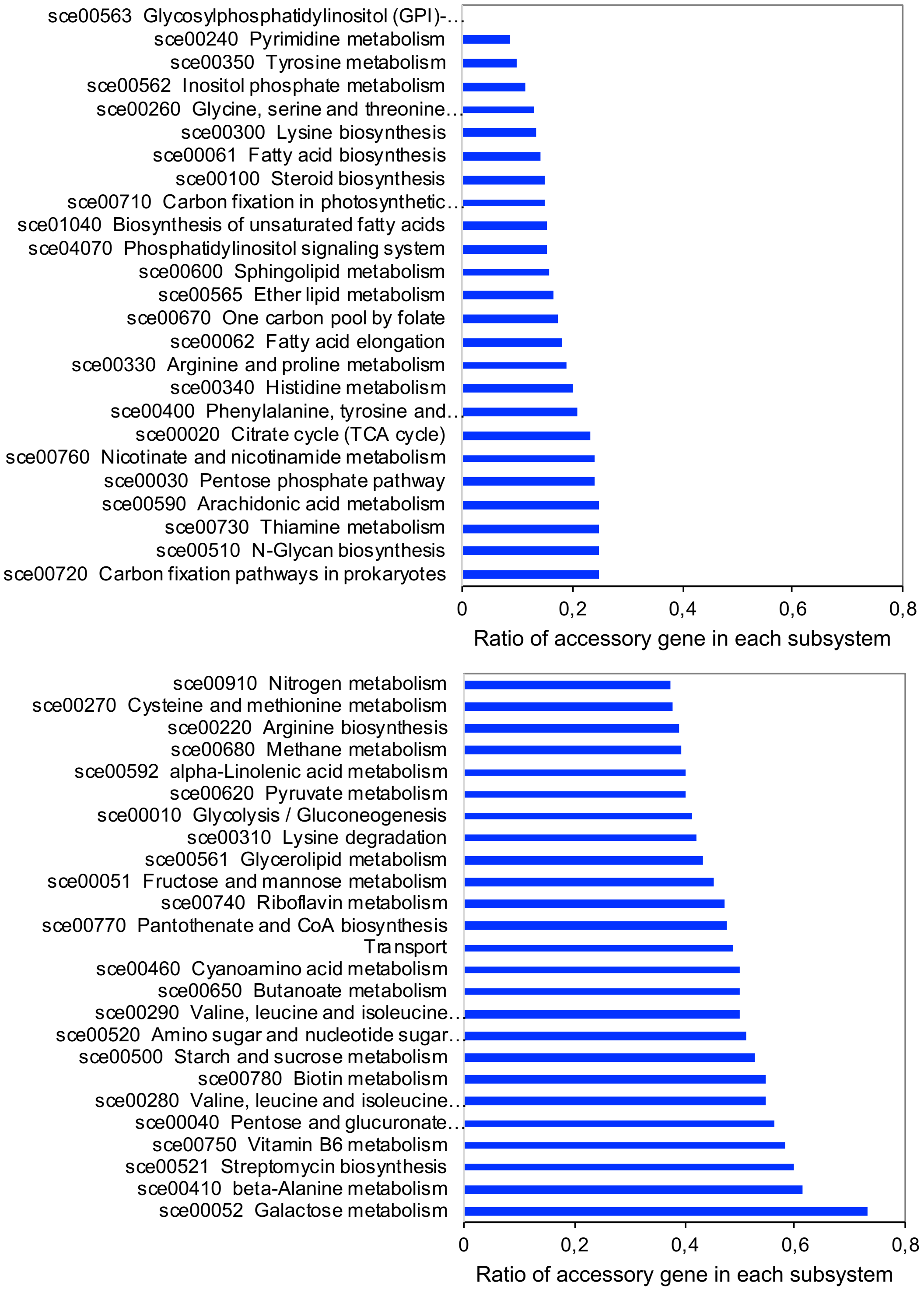

Supplementary Figure 8 Ratio of accessory genes in each subsystem. (a) 25 subsystems with lowest ratio of accessory genes. (b) 25 subsystems with highest ratios of accessory genes. In the above analysis, all the subsystems should have at least 8 genes.

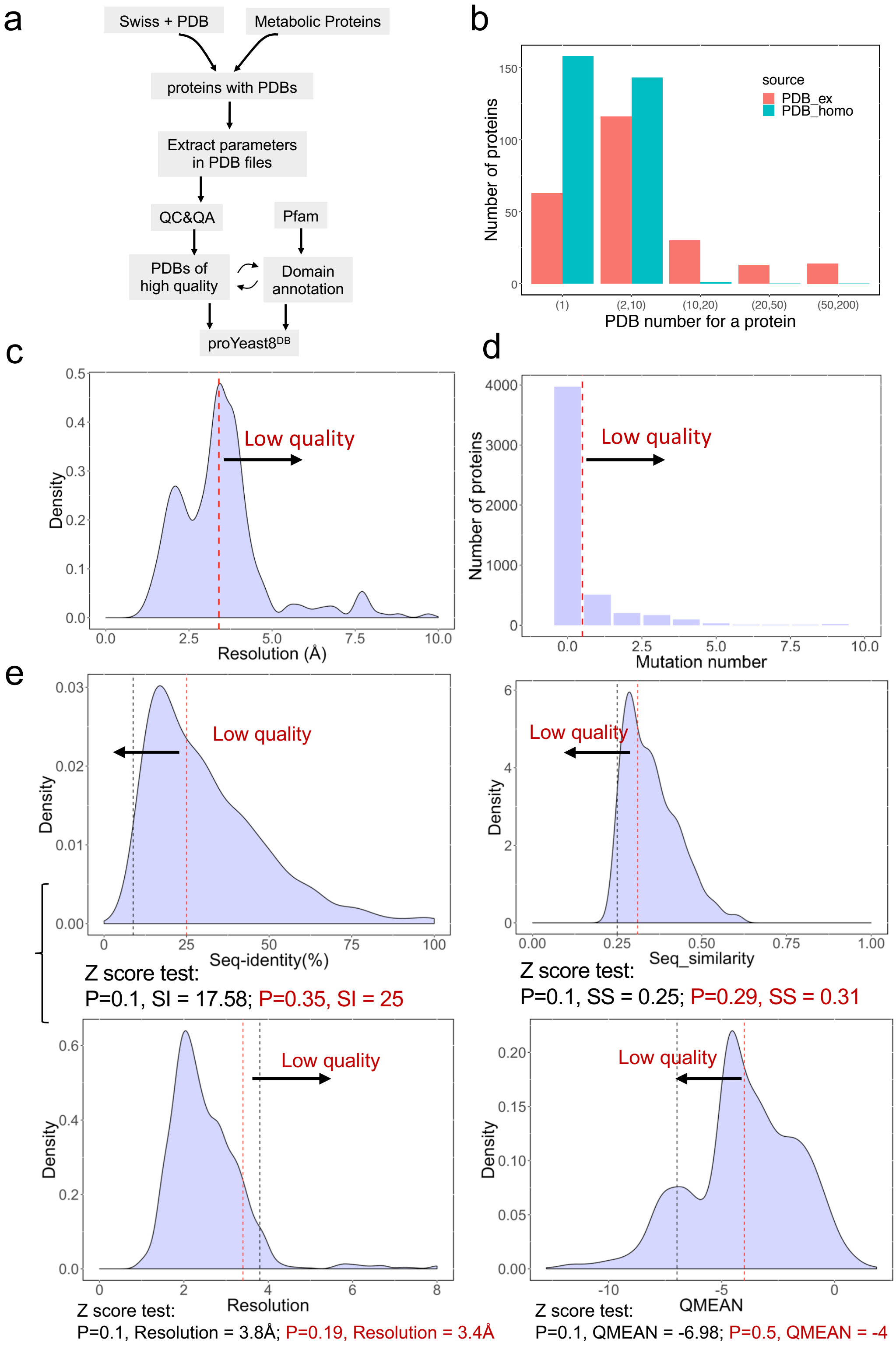

Supplementary Figure 9 Quality analysis for PDB files. (a) Pipeline to connect PDB files and domain information with proteins in Yeast8. (b) Summary of all the PDB_ex and PDB_homo for metabolic proteins. (c) Resolution distribution of all PDB_ex. (d) Statistical analysis in residue's mutation for all PDB_ex. (e) Quality analysis of PDB_homo for 910 proteins without PDB_ex

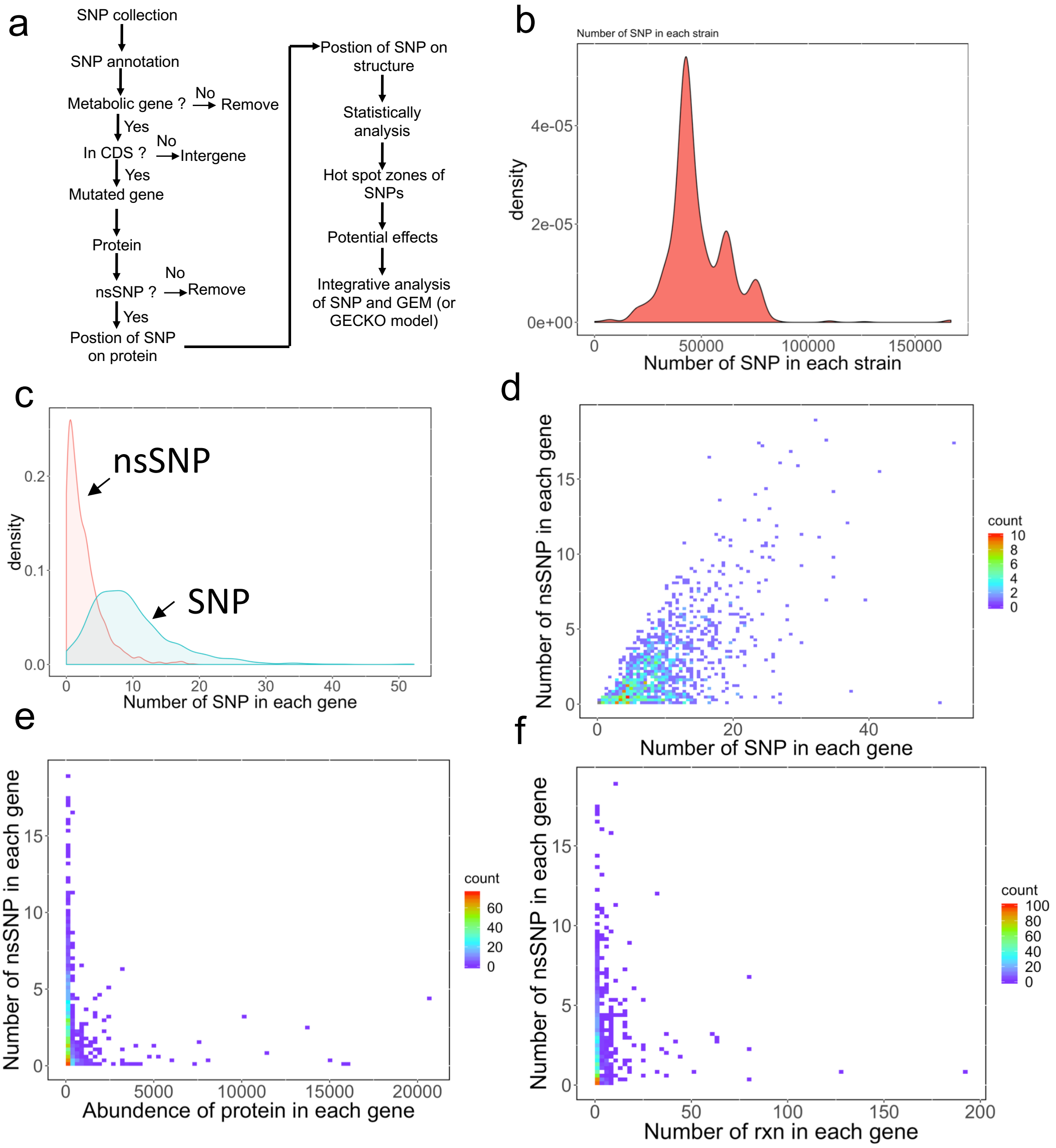

Supplementary Figure 10 SNP mapping analysis. (a) SNP analysis pipeline. (b) Distribution of SNP based on the 1,011 yeast strains. (c) Comparison between relative SNP and nsSNP number distribution. (d) Correlation between the relative SNP number and nsSNP number based on gene. (e) Correlation between relative nsSNP number and protein abundances. (f) Correlation between relative nsSNP number and rxn number connected with genes.

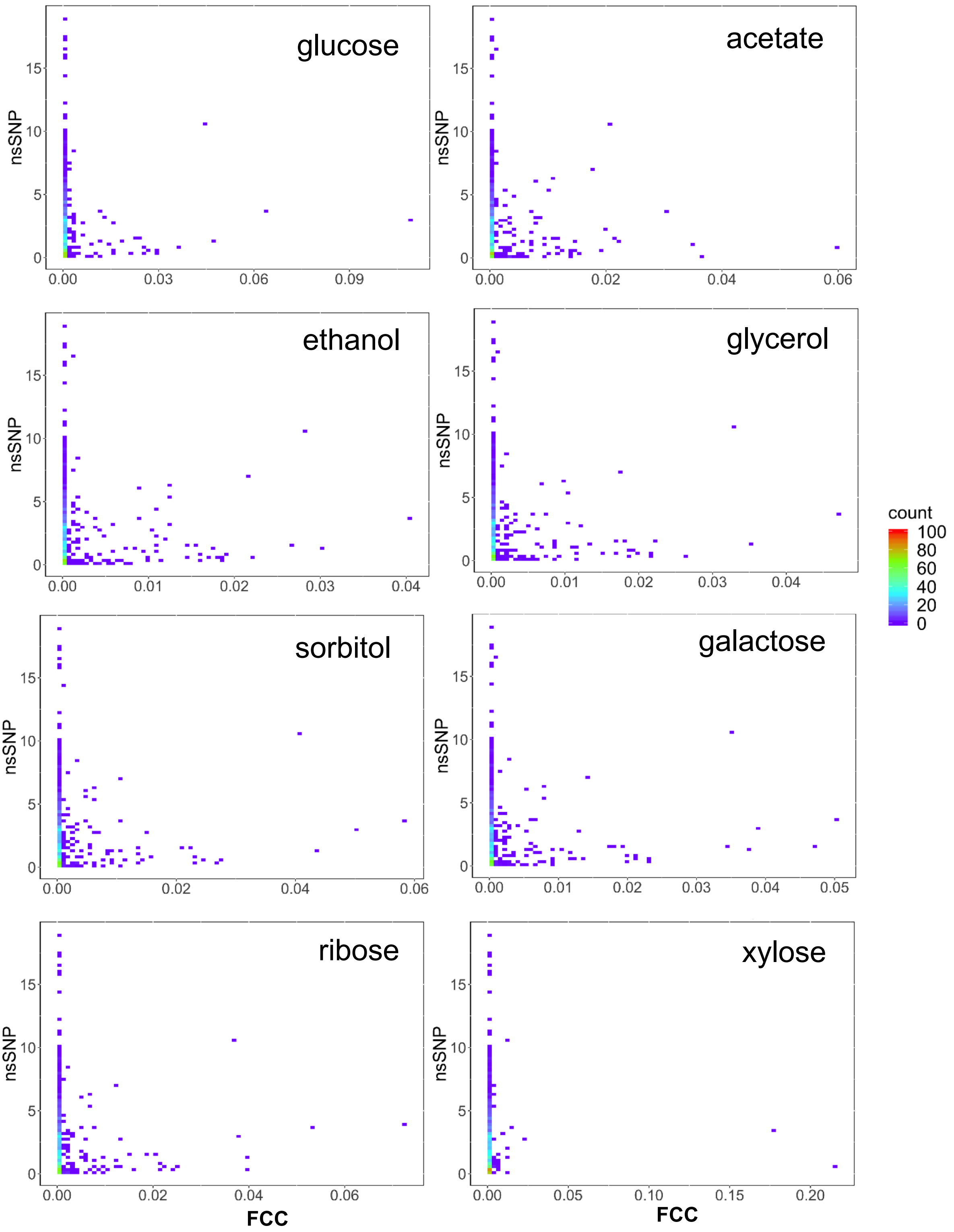

Supplementary Figure 11 Correlation between the relative nsSNP number and FCC obtained under different carbon sources (synthetic medium is used) with the growth as the objective function.

Supplementary Tables

Supplementary Table 1 Comparison between ecYeast7 and ecYeast8

Item	ecYeast7	ecYeast8
Number of reactions	6,741	8021
Number of metabolites	3,388	4145
Number of compartments	14	14
**Classification of reactions**		
Metabolic reactions matched with an enzyme(s)	3,239	3799
Metabolic reactions not matched with an enzyme	330	388
Transport reactions	1,674	1898
Metabolite exchange reactions	330	475
Arm reactions introduced for isozymes	404	501
Enzyme usages (treated as reactions)	764	963
**Classification of metabolites**		
Original metabolites	2,220	2680
Enzymes	764	963
Pseudo-metabolites introduced for isozymes	404	501
**Enzyme/reaction relationships**		
Complexes	226	305
Reactions with isozymes	373	399
Promiscuous enzymes	315	471

Supplementary Table 2 Summary of missing reaction in coreYeast8

Missing reaction	Missing rxn name	Composition name
r_0821	orotidine-5''-phosphate decarboxylase	CMP
r_0821	orotidine-5''-phosphate decarboxylase	UMP
r_0821	orotidine-5''-phosphate decarboxylase	dCMP
r_0821	orotidine-5''-phosphate decarboxylase	dTMP
r_0153	adenylosuccinate synthase	NAD
r_0913	phosphoribosylanthranilate isomerase	NAD
r_0153	adenylosuccinate synthase	NADH
r_0913	phosphoribosylanthranilate isomerase	NADH
r_0153	adenylosuccinate synthase	NADP(+)
r_0913	phosphoribosylanthranilate isomerase	NADP(+)
r_0153	adenylosuccinate synthase	NADPH
r_0913	phosphoribosylanthranilate isomerase	NADPH
r_4591	Zn(2+) transport	Zn(2+)
r_4589	Cu2(+) transport	Cu2(+)
r_4587	Ca(2+) transport	Ca(2+)
r_0061	3-isopropylmalate dehydrogenase	L-leucine
r_0659	isocitrate dehydrogenase (NADP)	L-lysine
r_0988	saccharopine dehydrogenase (NAD, L-lysine forming)	L-lysine
r_0913	phosphoribosylanthranilate isomerase'	L-tryptophan

Supplementary Table 3 Enrichment analysis of 24 genes with least nsSNP

Category	Term	p_value	FDR
GOTERM_BP_FAT	GO:0019438~aromatic compound biosynthetic process	2.57E-05	0.03244759
GOTERM_BP_FAT	GO:0009228~thiamin biosynthetic process	8.68E-05	0.10971671
GOTERM_BP_FAT	GO:0042724~thiamin and derivative biosynthetic process	0.000101	0.12761774
GOTERM_BP_FAT	GO:0006772~thiamin metabolic process	0.0001166	0.14732214
GOTERM_BP_FAT	GO:0042723~thiamin and derivative metabolic process	0.0001337	0.16890792
GOTERM_BP_FAT	GO:0006766~vitamin metabolic process	0.0002356	0.29742199
GOTERM_BP_FAT	GO:0006096~glycolysis	0.0003003	0.37899148
GOTERM_BP_FAT	GO:0044271~nitrogen compound biosynthetic process	0.0003371	0.42535266

Supplementary Table 4 Enrichment analysis of 32 genes with largest nsSNP

Category	Term	p_value	FDR
GOTERM_BP_FAT	GO:0044271~nitrogen compound biosynthetic process	0.00256127	3.31003994
GOTERM_BP_FAT	GO:0055114~oxidation reduction	0.00469175	5.98592708
GOTERM_BP_FAT	GO:0008652~cellular amino acid biosynthetic process	0.00861996	10.7412117
GOTERM_BP_FAT	GO:0006486~protein amino acid glycosylation	0.00981554	12.1437983
GOTERM_BP_FAT	GO:0043413~biopolymer glycosylation	0.00981554	12.1437983
GOTERM_BP_FAT	GO:0070085~glycosylation	0.00981554	12.1437983
GOTERM_BP_FAT	GO:0009309~amine biosynthetic process	0.0105323	12.9748602
GOTERM_BP_FAT	GO:0009101~glycoprotein biosynthetic process	0.01176064	14.3822228
GOTERM_BP_FAT	GO:0009100~glycoprotein metabolic process	0.01217451	14.8516572
GOTERM_BP_FAT	GO:0046394~carboxylic acid biosynthetic process	0.0200989	23.3937861
GOTERM_BP_FAT	GO:0016053~organic acid biosynthetic process	0.0200989	23.3937861

Supplementary Table 5 Enrichment analysis of proteins from strains of 'Wine'

Category	Term	p_value	FDR
GOTERM_BP_FAT	GO:0044271~nitrogen compound biosynthetic process	3.13E-05	0.0431749
GOTERM_BP_FAT	GO:0016053~organic acid biosynthetic process	4.22E-04	0.58032995
GOTERM_BP_FAT	GO:0046394~carboxylic acid biosynthetic process	4.22E-04	0.58032995
GOTERM_BP_FAT	GO:0006790~sulfur metabolic process	0.00122846	1.68143324
GOTERM_BP_FAT	GO:0006575~cellular amino acid derivative metabolic process	0.002422044	3.28997892
GOTERM_BP_FAT	GO:0006575~cellular amino acid derivative metabolic process	0.002422044	3.28997892
GOTERM_BP_FAT	GO:0009165~nucleotide biosynthetic process	0.002859394	3.87324182
GOTERM_BP_FAT	GO:0009260~ribonucleotide biosynthetic process	0.003763813	5.06906729
GOTERM_BP_FAT	GO:0034654~nucleobase, nucleoside, nucleotide and nucleic acid biosynthetic process	0.00403131	5.42010075
GOTERM_BP_FAT	GO:0034404~nucleobase, nucleoside and nucleotide biosynthetic process	0.00403131	5.42010075
GOTERM_BP_FAT	GO:0009259~ribonucleotide metabolic process	0.004118887	5.53476405
GOTERM_BP_FAT	GO:0008652~cellular amino acid biosynthetic process	0.004441768	5.95639822
GOTERM_BP_FAT	GO:0009309~amine biosynthetic process	0.00568197	7.55973724
GOTERM_BP_FAT	GO:0006749~glutathione metabolic process	0.006456195	8.54776575
GOTERM_BP_FAT	GO:0009145~purine nucleoside triphosphate biosynthetic process	0.007283372	9.59254123
GOTERM_BP_FAT	GO:0009206~purine ribonucleoside triphosphate biosynthetic process	0.007283372	9.59254123
GOTERM_BP_FAT	GO:0009144~purine nucleoside triphosphate metabolic process	0.007704569	10.1202733
GOTERM_BP_FAT	GO:0009205~purine ribonucleoside triphosphate metabolic process	0.007704569	10.1202733
GOTERM_BP_FAT	GO:0009201~ribonucleoside triphosphate biosynthetic process	0.008139947	10.6627702
GOTERM_BP_FAT	GO:0009199~ribonucleoside triphosphate metabolic process	0.008589616	11.2198851
GOTERM_BP_FAT	GO:0009142~nucleoside triphosphate biosynthetic process	0.009053681	11.7914549
GOTERM_BP_FAT	GO:0009141~nucleoside triphosphate metabolic process	0.012145557	15.5133327
GOTERM_BP_FAT	GO:0009066~aspartate family amino acid metabolic process	0.01450537	18.2553853
GOTERM_BP_FAT	GO:0022900~electron transport chain	0.017797719	21.9432926
GOTERM_BP_FAT	GO:0009152~purine ribonucleotide biosynthetic process	0.01850229	22.7121941
GOTERM_BP_FAT	GO:0006518~peptide metabolic process	0.01887879	23.1201843
GOTERM_BP_FAT	GO:0006091~generation of precursor metabolites and energy	0.019440868	23.7255579
GOTERM_BP_FAT	GO:0006091~generation of precursor metabolites and energy	0.019440868	23.7255579
GOTERM_BP_FAT	GO:0006091~generation of precursor metabolites and energy	0.019440868	23.7255579
GOTERM_BP_FAT	GO:0009150~purine ribonucleotide metabolic process	0.019957775	24.278377
GOTERM_BP_FAT	GO:0006164~purine nucleotide biosynthetic process	0.02305465	27.5133306
GOTERM_BP_FAT	GO:0006163~purine nucleotide metabolic process	0.025540287	30.0165462

Supplementary Table 6 Enrichment analysis of proteins from strains of 'Bioethonal'

Category	Term	p_value	FDR
GOTERM_BP_FAT	GO:0034605~cellular response to heat	0.04104534	41.6978615
GOTERM_BP_FAT	GO:0009408~response to heat	0.05459805	51.4590454
GOTERM_BP_FAT	GO:0009266~response to temperature stimulus	0.06423225	57.4555159
GOTERM_BP_FAT	GO:0009628~response to abiotic stimulus	0.1817105	92.4344347
GOTERM_BP_FAT	GO:0033554~cellular response to stress	0.21787911	95.7723179
GOTERM_BP_FAT	GO:0044265~cellular macromolecule catabolic process	0.33680707	99.4942341
GOTERM_BP_FAT	GO:0009057~macromolecule catabolic process	0.3626798	99.6969783
GOTERM_BP_FAT	GO:0044257~cellular protein catabolic process	0.45341606	99.9580409
GOTERM_BP_FAT	GO:0030163~protein catabolic process	0.47278102	99.9736271

Supplementary Table 7 CLUMPS analysis for different combinations of mutation from YJL052W

ID	cluster	p_value
1	V31;A73	0.0195
2	V31;A73;K24	0.0695
3	V31;A73;V70	0.0065
4	V31;A73;K24;V70	0.0247
5	V31;A73;K24;V70;S125	0.3977
6	V31;A73;K24;V70;S125;E248	0.7817
7	K24;V70	0.1089
8	S125;E248	0.6954

Supplementary Table 8 Summary of wrong metabolite annotation in yeast 7.6

Metabolite type	Number	Ratio in total metabolites
Metabolite with right chebiID	704	0.66
Metabolite with wrong chebiID	54	0.05
Metabolite with none chebiID	278	0.26
Metabolite with wrong full name	17	0.02
Metabolite with non-standard full name according to the chebiID	343	0.32
Metabolite with wrong formula	5	0.005
Metabolite with wrong charge	5	0.005
Metabolite with right keggID	584	0.55
Metabolite with wrong keggID	72	0.068
Metabolite without keggID	403	0.38

Supplementary Table 9 Main databases used in yeast GEM update from 7.6 to 8

Database	Web address	Main application
UniProt	https://www.uniprot.org/	gene annotation
KEGG	https://www.genome.jp/kegg/	gene annotation
BioCyc	https://biocyc.org/	gene annotation
SGD	https://www.yeastgenome.org/	gene annotation
Reactome	https://reactome.org/	gene annotation
TCDB	http://www.tcdb.org/	gene annotation
Rhea	https://www.rhea-db.org/	reaction and metabolite standardization
MetaNetX	https://www.metanetx.org/	reaction and metabolite standardization
BiGG	http://bigg.ucsd.edu/	reaction standardization
ChEBI	https://www.ebi.ac.uk/chebi/	metabolite check
YMDB	http://www.ymdb.ca/	metabolite check
EggNOG	http://eggnogdb.embl.de/#/app/home	gene annotation
ModelSeed	http://modelseed.org/genomes/	Reaction check
PABTHER	http://pantherdb.org/	Subsystem check

Supplementary Table 10 Metabolomics research summary for yeast

ID	Sources ID	Year	Metabolite_number	Reference
1	s1	2016	437	[1]
2	s2	2015	39	[2]
3	s3	2012	70	[3]
4	s4	2017	93	[4]
5	s5	2017	120	[5]
6	s6	2017	37	[6]
7	s7	2016	73	[7]
8	s8	2016	107	[8]
9	s9	2015	74	[9]
10	s10	2015	70	[10]
11	s11	2014	84	[11]
12	s12	2014	66	[12]
13	s13	2014	116	[13]
14	s14	2013	95	[14]
15	s15	2018	75	[15]
16	YMDB		870	[16]
17	s17	2018	38	[17]
18	s18	2018	74	[18]
19	s19	2017	89	[19]
20	s20	2017	36	[20]
21	s21	2017	45	[21]
22	s23	2017	143	[22]
23	s24	2007	88	[23]
24	s25	2013	21	[24]
25	s26	2017	26	[25]
26	s27	2016	50	[26]
27	s28	2018	51	[27]

Supplementary Table 11 Comparison of Yeast 8 with historical yeast GEMs

Model name	Gene	Reactions	Internal metabolites	Intracelluar compartments	Gene-associated reactions	Year
iFF708	619	1172	705	2	944	2003
iND750	750	1150	945	7	810	2004
iLL672	659	1095	670	2	872	2005
iMH805/775	805	1146	646	7	805	2006
iIN800	750	1150	945	2	810	2008
Yeast1	888	1857	1457	14	1407	2012
iMM904	904	1413	1064	7	1043	2009
Yeast4	924	1848	1868	15	1243	2012
iAZ900	901	1430	1070	7	1049	2010
Yeast5	918	1939	1484	15	1217	2012
iTO977	961	1293	1077	3	1046	2013
Yeast6	900	1888	1623	14	1180	2013
Yeast7.6	910	3498	2384	14	2310	2015
iSce926	926	3496	2223	14	2317	2015
Yeast8	1133	3949	2680	14	2507	2018

Supplementary Methods

Quality improvements for Yeast7.6

a) Correct the ChEBI IDs and KEGG IDs

Firstly, we systematically curated metabolite annotations in original Yeast7.6. After removing the comparTent information, there were a total of 1059 unique metabolites. 655 unique metabolites had KEGG IDs, 455 of which could be found in the KEGG database based on metabolite full names, while the remaining could not be found due to wrong or different molecular formulas. In total, 64 unique metabolites had duplicated KEGG IDs.

We then prepared three datasets used to correct the model metabolites information. Dataset1 contained detailed metabolite information from ChEBI database, which included the ChEBI IDs, formulas, charges, and names for each metabolite. Dataset2 contained the metabolite annotation from KEGG database, including the KEGG IDs, ChEBI IDs and names information for each metabolite. Dataset3 contains the merged metabolite annotation information from Dataset1 and Dataset2. In summary, the Dataset1 and Dataset3 contains the annotation of 134,614 terms while Dataset 2 contains the information of 18,107 items. Metabolites information from MetaNetX database was also merged with Dataset3 to fill in some missing information.

Based on the collected metabolite annotation information above, several in-house R scripts were used to check the metabolites annotation automatically from Yeast7.6. The correction process could be divided into four steps. Firstly, obtain the metabolites full name, formula, charge in Dataset3 using original ChEBI ID (KEGG ID); Secondly, obtain the metabolites ChEBI ID and KEGG ID (new) in Dataset1 and Dataset2 using original full name mapping; Thirdly, compare old and new information in ChEBI ID, KEGG ID, full name, formula, charge respectively for each metabolite; Lastly, based on the correct ChEBI ID (or KEGG ID) information, obtain all other information (like BiGG ID, structure information, database link). The corrected metabolite information can be found in Supplementary Table 8. These wrong metabolites information had been found and were corrected to improve the model quality in metabolites annotation.

b) Add subsystem automatically for yeast GEM

Yeast7.6 had no subsystems annotation. An in-house Matlab script was adopted to find subsystem information from KEGG database based on gene annotation. If no subsystem information was available for particular gene from KEGG, the related subsystem information from BioCyc and Reactome was used. To correct the above subsystem information, subsystems for the reactions in yeast GEM were further queried based on the reaction ID mapping from KEGG database. In the above procedures, one reaction could have several different subsystems based on the functional annotation from KEGG database.

c) Refine subsystem information for map of yeast GEM

For the map of Yeast8, a unique subsystem was given for each reaction. To refine and simplify the subsystems in step b, the subsystems from different models (Yeast7.6, Recon3D, HMR2, iTO977 and iMM904) and databases (MetaNetX, BioCyc, Reactome and KEGG) were compared and merged in aspects of reactions' (or genes') metabolic function. Subsystem information from iTO977 and iMM904 was borrowed when it is absent for part of reactions in yeast GEM. For reactions still absent in the subsystem definition, the manual check of gene annotation in above databases will be used to find the corresponding subsystems. As one reaction could have a different subsystem information based on KEGG databases, the following procedures were then used to refine the subsystem information: (1) Remove the more general subsystem information, like carbon metabolism; (2) Remove the wrong subsystems according to the annotations of metabolites from the corresponding reactions; (3) Remove the subsystems linked with no more than three reactions.

It is oftentimes not easy to define unique subsystem information; reactions such as amino acid metabolism is one example of this phenomenon. A more general metabolism subsystem can be used to cover small subsystems with few reactions. Based on the correction process, moreover, each reaction now has clear subsystem information and can be used to draw the yeast map.

d) Add reference ID for each reaction
We collected the reference ID information from the MetaNetX database and merged it with Yeast7.6. Roughly 30% of reactions could find BiGG IDs and MetaNetX IDs. Only 15% of reactions, however, could find KEGG IDs.

Adding new GPRs by merging Yeast7.6, iTO977 and iSce926
We found that the two historical yeast models (iTO977 and iSce926) contained more genes compared to Yeast7.6, which only contained 909 genes. As the iSce926 was updated based on Yeast7.6, 14 new genes from iSce926 were firstly merged into Yeast7.6 while the other four genes were not added based on the gene functional annotation. When compared with iTO977, 104 genes were not included in Yeast7.6. After an initial check, we found that some GPRs (like L-serine => NH_3 + pyruvate is connected with 'YIL167W or 'YIL168W') were wrong, thus we compared the gene annotations systematically in different databases before merging them into Yeast7.6.

Adding new GPRs based on gene function annotation in 5 databases
a) Compare the gene annotation from main databases
As a model organism, the reference yeast strain-*Saccharomyces cerevisiae* S288C has quite a detailed genome annotation from several popular genome and model databases, which include Reactome, BioCyc, KEGG, NCBI, SGD, and UniProt (Supplementary Table 9). Among them, SGD is specially designed for *S. cerevisiae* and contains very detailed gene function annotation information based on literature reports. To improve the coverage of genes for yeast GEM, the latest gene annotation of *S. cerevisiae* in SGD, UniProt and KEGG were collected. At the same time, the reactions connected with the gene from Reactome, KEGG, BioCyc, and UniProt were also collected. All reactions were a foundation for the researchers of this study to choose a GPRs for Yeast8. As shown in Supplementary Fig.3a, the protein number annotated by UniProt is the largest. By comparison, total 5819 proteins could be found in all four databases, which can be regarded as 'high quality' for the genes function existent in *S. cerevisiae* S288C genome.

b) Procedures to find new GPRs
Different strategies were employed to extract the new GPRs from the main databases. For the UniProt and SGD, the gene list in Yeast7 was compared to all yeast genes in these two databases. Based on the genome annotations, furthermore, we can obtain the function annotation of genes, including the EC number and the catalysed reactions. Based on EC number, we could find the standard reaction formulas from Rhea, Brenda, or MetaNetX. The new genes were obtained in a similar method using the KEGG database. Next, KO id was given for each gene based on KEGG genome annotation. With KO mapping, the related reactions and enzymes connected with genes could be obtained and would further formulate into pairs of new genes (proteins) and reactions.

Reactome[28] and BioCyc[29] contain detailed metabolic reactions f *S. cerevisiae* S288C, both of which are excellent sources for discovering new GPRs. Based on BioCyc annotation, we can find the gene-reaction relations without EC number and gene-EC number-reaction relations. A detailed procedure was designed for the Reactome database to extract the related new GPRs. Firstly, we downloaded three kinds of mapping data from Reactome, including geneID (NCBI)-protein-reaction, gene-protein-pathway, and the pathway-pathway relation including the hierarchical relationships of pathways defined in Reactome, for example, the metabolism pathway could contain a lot of sub-pathway related to the detailed cell metabolism activities. Based on pathway hierarchical relationships, we have been able to obtain gene-protein-reactions which belong to the metabolic

pathway, as well as the transport pathway of small molecules. We are in the process of downloading NCBI genome annotations from the NCBI database for further research. This database contain mapping between the NCBI geneID and gene systematic name. Using the mapping, we can finally obtain the standard geneID-protein-reactions relation based on Reactome and can compare results with other databases.

c) New GPRs comparison across different sources
In summary, total 977 unique genes were obtained based on all the gene annotation in KEGG, SGD, UniProt, Reactome and BioCyc. By comparison, 709 genes were found from UniProt while only 119 genes were obtained from Reactome databases. As shown in Supplementary Fig. 3b, most of genes could be found in at least two databases and a few of them only existed in one database. To ensure its quality, the chosen genes should be present in at least two databases. Before obtaining the potential new genes, all genes were classified into three types: "Yes", "Not Sure", and "No", based on 26 kinds of annotation information, such as the related function annotation, EC, and subsystem.

In this step, 308 genes belong to the "Yes" category, while 315 genes belong to the "Not Sure" category, and 353 genes belong to the "No" category. Thus, only 308 genes will enter into the next step. As shown in Supplementary Fig. 3d, of these 308 new genes, 305 of them could be found in KEGG, SGD, UniProt, and NCBI. During this step, the gene annotation in SGD and UniProt were manually checked with the related reactions from different databases. We only chose reactions which were consistent with the reliable evidence of gene function annotation in SGD and UniProt.

d) Quality improvement of new GPRs
Once the new genes or new GPRs are prepared, the quality improvement will be done for all of them using the mentioned 'general procedures' (Method). If the gene is connected with reactions which are existent in Yeast7, it will be merged into the old GPRs. Otherwise a new GPR will be merged into Yeast7. All the referent IDs of reactions and metabolites annotation will be obtained based on 'general procedures'. Each newly added reaction will be charged and mass balanced. In subsystem check, potential new genes are distributed among 75 subsystems, among which 14 subsystems can be merged with the present subsystem in Yeast7 according to their function, while 45 subsystems can be found directly from Yeast7. For newly added subsystem with only one or two reactions, we put it them into subsystem "Miscellaneous". After this, we mainly obtained several new subsystems compared with the Yeast7, like methylglyoxal metabolism, lipoic acid metabolism, and so on.

In this round of model update, 48 original GPRs in Yeast7 were updated by merging with the new genes. Meantime, 183 new reactions with 163 genes were added into Yeast7. These updates have improved the yeast GEM version from Yeast8.1 to Yeast8.2. The detailed description can be found in GitHub of yeast GEM (https://github.com/SysBioChalmers/yeast-GEM).

Improve the gene coverage for transport reactions based on gene annotation in TCDB
In yeast model, 174 of 1029 transport reactions did not have gene relations. We firstly summarized all the transport reactions with gene association from the isacce[30] model and Yeast8. Besides this, we re-annotated the S288C genome with EggNOG web services. In the annotation, the BiGG reaction ID was obtained for part of the genes, whereby the gene connected with transport reactions was chosen. Based on the above three data sources, a gene-transported metabolites matrix was established. The matrix contains the gene name, the connected reaction, the main transported metabolite, the participated metabolites (like H or ATP), along with the MNXID (metabolite ID in MetaNetX database) for the main transported metabolites.

On the other hand, the yeast transporter protein annotation from TCDB database was carefully checked. The gene with TCDB id was firstly chosen, followed by detailed gene annotations from the TCDB. Furthermore, the detailed annotation from the 5 main databases (Reactome, BioCyc,

KEGG, SGD, and UniProt) was also added for each gene. With all the above information, we can find all the metabolites transported by the reliable proteins from yeast genome. According to the gene-metabolite matrix, the transporter proteins were mapped onto the transport reactions without gene association. Moreover, the protein compartment and subunit information were further checked before adding gene relations for the aimed transport reactions. In this step, the number of transport reactions with genes was improved from 174 to 275.

Adding new reactions based on Biolog experiments
a) Biolog Phenotype MicroArrays experiment
The growth of *S. cerevisiae* S288C and CEN-PK 113.7D on different substrates were performed on Biolog Phenotype MicroArrays, which includes 190 carbon sources, 95 nitrogen sources, 59 phosphorus sources, and 35 sulfur sources. 20 carbon sources tested can be utilized by *S. cerevisiae* S288C, and eight more carbon sources by *S. cerevisiae* CEN-PK 113.7D. As for nitrogen sources, *S. cerevisiae* S288C has the ability to utilize 40 substrates, while *S. cerevisiae* 113.7D can utilize an additional four substrates. *S. cerevisiae* s228c and CEN-PK 113.7D can both utilize 48 phosphorous sources and 19 sulfur sources (Supplementary Data 1).

b) Model prediction
The results above could be used to evaluate model predictions in substrates usage. The Matlab function 'SubstratUsage.m' was developed to detect whether each substrate can be utilized *in silico*, and if the substrate cannot be utilized, the function would suggest the reason for that by stating which kind of reaction is missing. This function automatically adds an exchange reaction for that substrate and sets the lower bound of that exchange reaction to -10 mmol $(gDW)^{-1}$ h^{-1} for carbon and nitrogen substrate and -1000 mmol $(gDW)^{-1}$ h^{-1} for sulfur and phosphorus substrates. Minimal media was used in this prediction and growth was set as the objective function. It is regarded as no growth if the predicted growth rate is less than or equal to 10^{-8} h^{-1}. We then compared the predicted growth phenotype with experimental results and all results could be classified into: (1) G/G-*in vivo* growth/*in silico* growth; (2) NG/NG-*in vivo* no growth/*in silico* no growth; (3) G/NG-*in vivo* growth/in *in silico* no growth; and (4) NG/G-*in vivo* no growth/*in silico* growth.

c) Add new reactions into yeast GEM based on *in silico* and *in vivo* results
For the NG/G condition, we leave as it is as there may be some regulatory factors determining the inconsistency. While for those inconsistencies in G/NG condition, it can be reconciled by filling gaps in the model. The Matlab function 'SubstratUsage.m' was firstly used to check whether this substrate existed in the model, if yes, then such an inconsistency is mainly due to the lack of transport reactions in the present model. If the substrate did not exist in the model, then the model lacked the related pathways for the substrate metabolism. To reconcile the inconsistency caused by the missing of the related transport reactions, we extracted essential transported reactions from MetaNetX database to connect that substrate with yeast GEM. As for the inconsistency caused by missing pathways, the reactions including the related substrates from MetaNetX were extracted. In order to add a minimal amount of reaction without genetic confidence, the followed criteria were used to screen essential reactions: (1) Introduce the least number of new metabolites; (2) For carbon substrates, if it is sugar-derived, add sugar degradation reaction. If not, proceed to add the reaction that can link this substrate to central metabolites; (3) For nitrogen substrates, add degradation reaction to ammonium or N-containing central metabolites; (4) For sulfur substrates, add a degradation reaction to a simple S-containing metabolites (sulfite, sulfate, hydrogen sulfide, or S-containing central metabolites; (5) For phosphorus substrates, add a degradation reaction to phosphate.

The new reactions were carefully chosen to fill the gap in the above two conditions. Since these reactions are simply for modelling purposes, the confidence score for these reactions was set as 1[31]. To further reduce the inconsistency, the reversibility of four reactions in the model were corrected

for a better prediction. Firstly, for thymidine and thymidine derived substrate usage, the reversibility of reaction r_2075 (transporting thymidine) was changed to be reversible so it is now consistent with the reaction R_00573 in the model of milk yeast *Kluyveromyces lactis*[32]. Secondly, for N-acetyl-L-glutamate to be utilized as a nitrogen source, the reversibility of reaction r_0761 was changed to be reversible according to MetaCyc reaction N-ACETYLTRANSFER-RXN. Thirdly, for acetoacetate to be utilized as carbon source, the reversibility of reactions r_0104 and r_0103 was changed to be reversible according to MetaCyc reaction ACETYL-COA-ACETYLTRANSFER-RXN.

During this update, total 225 new reactions and 148 new metabolites were added into yeast GEM. The model prediction results are shown in Supplementary Fig. 4b. Model prediction accuracy for substrate usage were increased from 63.4% to 81.5%.

Adding new reactions based on metabolomics mapping
We collected metabolomics data from 26 papers to form a metabolites pool with 1774 metabolites (Supplementary Table 10). The standard identifiers from KEGG and ChEBI database were found automatically for all the metabolites. As a result, 436 metabolites without any standard annotation were filtered out. With manual check, it can be found that 493 metabolites existed in the model and 766 metabolites were confirmed as new metabolites. Next, only metabolites originated from at least two reports (including the recording in YMDB) were used and finally 82 metabolites were prepared for the new update of yeast GEM.

To connect these new metabolites with yeast GEM, 852 reactions connected with those metabolites from MetaNetX database were extracted. In order to introduce the least number of new metabolites except those 82 metabolites into the model, the reactions that contain more than two extra new metabolites were removed and 177 reactions were kept for further analysis. Reactions that catalysed by enzymes or EC number existing in the yeast genome annotation were chosen firstly (this included 32 reactions). Since we did not want to introduce additional dead-ends into the model, we only chose new metabolites which can be produced and consumed in the new model. Therefore, only 21 reactions for eight new metabolites were added into yeast GEM.

Adding new reactions based on gap filling
After several rounds of update, some dead-ends metabolites were also added into the model. Here, to avoid adding a lot of reactions without genetic information, gap-filling was only conducted for those metabolites which lack the related transport reactions. Function “metsincomps.m” and “MissingTransDeadEnd.m” were used to identify dead-end metabolites which can be fixed by adding a transport reaction. We then extracted transport reactions for those metabolites from MetaNetX database. As a result, 37 new transport reactions were found to reduce 45 dead end metabolites in the model.

Improving the biomass equation
Some reactions and pathways in our model do not carry flux. The NADH synthesis pathway, for example, does not carry any flux when biomass is set as the objective function. This is because NADH and NAD form a cycle in original model. Besides, there are no ions in the biomass, which means that all ions transport reactions do not carry flux either. Based on this, the original biomass equation was expanded to include cofactors and ions. We compared all biomass components from historical yeast models (Supplementary Table 11), and collected information from several papers to estimate which metabolites should be included[33-38]. As for cofactors, NADH, NAD, NADPH, NADP, and coenzyme A, THF, TDP, and FAD were newly added. Coefficients for these metabolites were calculated from cellular concentrations from reference[33-38]. As for ions, calcium, chloride, cooper (II), iron (II), manganese (II), zinc (II), potassium (II), sodium (II), and magnesium (II) were newly added. The coefficients for those newly added metabolites were calculated from references[33-]

[36, 39, 40] along with the recorded cellular concentration in the YMDB database. A Matlab function "scale_BioMass.m" was used to re-scale biomass composition and re-fit the growth-associated maintenance energy (GAM), in order to make sure the biomass weight is equal to 1 gram and physiological kinetic parameters from chemostat experiments could be predicted well.

Comparison with historical yeast models

The yeast GEM has developed for 15 years. As shown in Supplementary Table 11, along the time, the number of genes, reactions, and metabolite numbers have increased consistently since iFF708. For Yeast8, after the systematic update, the numbers of genes and reactions undergo a significant increase compared with Yeast7.

Protein existence analysis based on proteomics data

We downloaded proteomics data from PaxDb (https://pax-db.org). With geneID mapping, we can obtain the abundance for all newly added genes. The result showed that 98.34% of new genes have protein evidence and only three genes (YAR069W-A, YHR214W-F, and YPL096C-A) were not detected based on PaxDb. We also obtained gene annotation scores from UniProt database. 208 genes can be verified at least in the protein level and only four have no omics evidence.

Supplementary Note 1

Pan-genome re-annotation

To roughly estimate new proteins in non-reference ORFs (nrORFs) compared with reference genome, the blast analysis of nrORFs using Diamond[41] against UniProt, TCDB, and BiGG reference protein database [42] were conducted respectively. It can be found that as the pidentity increased, the protein number with new hit not from reference genome decreased significantly (Supplementary Fig. 6c). It can further be found that if the pidentity is set at 75%, there are a few new hits for those genes from nrORFs, which means that the metabolism of yeast is quite conservative and new proteins can be quite limited.

We used the blast analysis, the gene annotation of KEGG web service[43] and EggNOG web service[44] to check the original ortholog relation in the article. Among them, 25 nrORFs' ortholog genes were updated based on the original ortholog gene relation from article[45]. Three nrORFs (88-augustus_masked-125-YCE, 346-augustus_masked-779-BSG_4, 790-augustus_masked-AMH_5-6637) have different ortholog genes based on at least two methods of blast, KEGG, and EggNOG service, thus needing further check. In total, 771 panID have reliable ortholog genes from *S. cerevisiae* S288C based on KEGG, eggnog, and blast analysis. To further evaluate the ortholog gene relations qualitatively, the BBH analysis between nrORFs and *S. cerevisiae* S288C genomes was used. The best hit in BBH analysis with pidentity larger than 80% was finally chosen, among which, 208 nrORFs could find the corresponding ortholog genes from Yeast8 (*S. cerevisiae* S288C genomes).

Through the pan-genome annotation, 49 new RxnIDs with 13 panIDs from KEGG and 7 new RxnIDs with 4 panIDs from EggNOG were prepared for panYeast8. By comparing the gene list from Yeast8 and pan-genome, it could be found that about 33 genes in Yeast8 are non-existent in the pan-genome. This is due to the fact that in the pan-genome definition, some genes with duplicated function with their ortholog genes from *S. cerevisiae* S288C have collapsed. As a result, the panID was given for the 29 collapsed gene based on the ortholog genes which have the panID. For the genes without ortholog genes, the blast analysis was used to find the best hit from pan-genome.

In silico growth simulation of coreYeast8

After the reconstruction of the strain specific GEMs (ssGEMs), we formulated the coreYeast8 based on shared reactions, metabolites, and genes for all yeast strains. The coreYeast8 contains 3895 reactions, 2666 metabolites, and 892 genes. We evaluated the metabolic capacity of coreYeast8 based on minimal media and found that part of biomass compositions cannot be synthesized, which include: dCMP, dTMP, CMP, UMP, NADH, NADPH, NADP+, and NAD+, along with several kinds of amino acids. Also, by comparing differences between the coreYeast8 and ssGEMs, it can be found that 'r_4591' for Zn^{2+} transport, 'r_4587' for Ca^{2+} transport and 'r_4589' for Cu^{2+} transport are absent in the coreYeast8. Other missing reactions in coreYeast8 can be found in Supplementary Table 2.

In silico maximal yield analysis of 20 amino acids using the simulation of 1011 strain specific models

The amino acid synthesis pathways are well conserved among these 1011 yeast stains. The difference in theoretical maximum amino acid yields are due to two main reasons. Firstly, some strains with low maximal amino acids yields use fermentation instead of respiration to produce energy for amino acid synthesis, because they don't have all essential subunits for respiration complexes. Those strains should not be able to use non-fermentable carbon sources such as ethanol and glycerol for growth as phenotypes if the respiration is impaired as our model indicated. According to our models, *S. cerevisiae* AAH uses fermentation to produce energy for amino acid

synthesis and has low maximal amino acid yields. This strain grows poorly on the medium with ethanol (relative growth rate: 0.00562701) and glycerol (relative growth rate: 0.00617284) as the main carbon sources according to literature[45].
Secondly, a few synthesis reactions for some important amino acid are missing in several strains, resulting in non-production of the corresponding amino acids. For example, in *S. cerevisiae* BLT and AHG, YHR208W or its ortholog 179-augustus_masked-2806-CPI_4 (valine transaminase, mitochondrial) in the last step of valine synthesis are missing, so the maximal yield of valine for those strains is decreased to zero. Similarly, YNL220W (adenylosuccinate synthase) related for histidine synthesis is missing in strain *S. cerevisiae* ABM, so the related maximal histidine yield is decreased to zero in our simulation. There is only one strain *S. cerevisiae* SACE_GAV, which did not have the gene YDR007W (phosphoribosylanthranilate isomerase) in the third step in tryptophan biosynthesis, so the related maximal yield for tryptophan is decreased to zero. These results together may indicate the auxotrophic phenotypes existing in part of these 1011 strains.

Supplementary Note 2

Refining the quality of protein 3D structures

In this work, there have been a total number of 1234 metabolic genes (the gene list in Yeast8 and a few other metabolic genes not added into Yeast8). There are 910 proteins without experimental PDB files (PDB_ex). A total of 1486 homology PDB files (PDB_homo) can be found for these 910 proteins. 299 of proteins were connected with over two PDB files. If the cut-off value for sequence identity, sequence similarity, resolution, and QMEAN are 17.58, 0.25, 3.8Å, and -6.98 respectively (*P* value = 0.1 in zero score test), 183 PDB_homo with QMEAN are smaller than -6.98, 339 PDB_homo with SI smaller than 17.58%, 33 PDB_homo with SS smaller than 0.25, and 364 PDB_homo with Resolutions larger than 3.8Å. However, as stated in the SWISS-MODEL database, the PDB_homo with a QMEAN larger than -4 can be accepted in the quality. On the other hand, for the homology model, the quality of PDB_homo with SI ≥ 0.25 can be accepted. Therefore, it seems that the *P* value of 0.1 is relatively large as the cut-off. To ensure high quality of chosen PDB_homo for further analysis, the critical parameters were reset as follows: QMEAN ≥ -4, SI ≥ 0.25, SS ≥ 0.31 and Resolution ≤ 3.4Å. With new cut-off, 534 PDB_homo for 444 proteins can be regarded as high quality. The other PDB_homo will be ranked based on their QMEAN values.

There are 3332 PDB_ex for all the 319 proteins existed in yeast GEM. Only about 60 proteins have the single PDB files. We further check whether there exist gaps in the PDB_ex files. As a result, we found that 44 PDB_ex with mutation (pidentity <100) and 20 PDB_ex with lower resolution (≥3.4Å). So for these PDB_ex, they are replaced of by the corresponding PDB_homo from the SWISS-MODEL database. Finally, the PDB_ex for 162 proteins could be used in the protein structure mapping analysis.

Supplementary References

1. Raguz Nakic, Z., Seisenbacher, G., Posas, F. & Sauer, U. Untargeted metabolomics unravels functionalities of phosphorylation sites in *Saccharomyces cerevisiae*. *BMC Syst Biol* **10**, 104 (2016).
2. Teoh, S.T., Putri, S., Mukai, Y., Bamba, T. & Fukusaki, E. A metabolomics-based strategy for identification of gene targets for phenotype improvement and its application to 1-butanol tolerance in *Saccharomyces cerevisiae*. *Biotechnol Biofuels* **8**, 144 (2015).
3. Bergdahl, B., Heer, D., Sauer, U., Hahn-Hagerdal, B. & van Niel, E.W. Dynamic metabolomics differentiates between carbon and energy starvation in recombinant *Saccharomyces cerevisiae* fermenting xylose. *Biotechnol Biofuels* **5**, 34 (2012).
4. Schultz, M.C. et al. Impact of Low-Intensity Pulsed Ultrasound on Transcript and Metabolite Abundance in Saccharomyces cerevisiae. *J Proteome Res* **16**, 2975-2982 (2017).
5. Kim, J. & Kim, K.H. Effects of minimal media vs. complex media on the metabolite profiles of *Escherichia coli* and *Saccharomyces cerevisiae*. *Process Biochemistry* **57**, 64-71 (2017).
6. Hammerl, R., Frank, O. & Hofmann, T. Differential Off-line LC-NMR (DOLC-NMR) Metabolomics To Monitor Tyrosine-Induced Metabolome Alterations in *Saccharomyces cerevisiae*. *Journal of agricultural and food chemistry* **65**, 3230-3241 (2017).
7. Nugroho, R.H., Yoshikawa, K., Matsuda, F. & Shimizu, H. Positive effects of proline addition on the central metabolism of wild-type and lactic acid-producing *Saccharomyces cerevisiae* strains. *Bioprocess Biosyst Eng* **39**, 1711-1716 (2016).
8. Kim, S. et al. Elucidation of ethanol tolerance mechanisms in *Saccharomyces cerevisiae* by global metabolite profiling. *Biotechnol J* **11**, 1221-1229 (2016).
9. Nugroho, R.H., Yoshikawa, K. & Shimizu, H. Metabolomic analysis of acid stress response in *Saccharomyces cerevisiae*. *J Biosci Bioeng* **120**, 396-404 (2015).
10. Wang, X. et al. Increasing proline and myo-inositol improves tolerance of *Saccharomyces cerevisiae* to the mixture of multiple lignocellulose-derived inhibitors. *Biotechnol Biofuels* **8**, 142 (2015).
11. Hashim, Z., Teoh, S.T., Bamba, T. & Fukusaki, E. Construction of a metabolome library for transcription factor-related single gene mutants of *Saccharomyces cerevisiae*. *J Chromatogr B Analyt Technol Biomed Life Sci* **966**, 83-92 (2014).
12. Kawase, N., Tsugawa, H., Bamba, T. & Fukusaki, E. Different-batch metabolome analysis of *Saccharomyces cerevisiae* based on gas chromatography/mass spectrometry. *J Biosci Bioeng* **117**, 248-255 (2014).
13. Chumnanpuen, P., Hansen, M.A., Smedsgaard, J. & Nielsen, J. Dynamic Metabolic Footprinting Reveals the Key Components of Metabolic Network in Yeast *Saccharomyces cerevisiae. Int J Genomics* **2014**, 894296 (2014).
14. Kim, S. et al. Evaluation and optimization of metabolome sample preparation methods for *Saccharomyces cerevisiae*. *Anal Chem* **85**, 2169-2176 (2013).
15. de Ruijter, J.C., Koskela, E.V., Nandania, J., Frey, A.D. & Velagapudi, V. Understanding the metabolic burden of recombinant antibody production in *Saccharomyces cerevisiae* using a quantitative metabolomics approach. *Yeast* **35**, 331-341 (2018).
16. Ramirez-Gaona, M. et al. YMDB 2.0: a significantly expanded version of the yeast metabolome database. *Nucleic Acids Research* **45**, 440-445 (2017).
17. Lyu, X., Ng, K.R., Mark, R., Lee, J.L. & Chen, W.N. Comparative metabolic profiling of engineered *Saccharomyces cerevisiae* with enhanced flavonoids production. *Journal of Functional Foods* **44**, 274-282 (2018).
18. Pan, D., Lindau, C., Lagies, S., Wiedemann, N. & Kammerer, B. Metabolic profiling of isolated mitochondria and cytoplasm reveals compartment-specific metabolic responses. *Metabolomics* **14**, 59 (2018).
19. Bu, X., Sun, L., Shang, F. & Yan, G. Comparative metabolomics profiling of engineered *Saccharomyces cerevisiae* lead to a strategy that improving beta-carotene production by acetate supplementation. *PLoS One* **12**, e0188385 (2017).
20. Mendes, I. et al. Integrating transcriptomics and metabolomics for the analysis of the aroma profiles of *Saccharomyces cerevisiae* strains from diverse origins. *BMC Genomics* **18**, 455 (2017).
21. Dong, Y., Hu, J., Fan, L. & Chen, Q. RNA-Seq-based transcriptomic and metabolomic analysis reveal stress responses and programmed cell death induced by acetic acid in *Saccharomyces cerevisiae*. *Sci Rep* **7**, 42659 (2017).
22. Buettner, F. et al. Non-targeted metabolomic approach reveals two distinct types of metabolic responses to telomerase dysfunction in *S. cerevisiae*. *Metabolomics* **13**, 53 (2017).
23. Castrillo, J.I. et al. Growth control of the eukaryote cell: a systems biology study in yeast. *J Biol* **6**, 4 (2007).
24. Ibanez, A.J. et al. Mass spectrometry-based metabolomics of single yeast cells. *Proc Natl Acad Sci U S A* **110**, 8790-8794 (2013).
25. Shen, Y. et al. Deep functional analysis of synII, a 770-kilobase synthetic yeast chromosome. *Science* **355** (2017).

26. Puig-Castellvi, F., Alfonso, I., Pina, B. & Tauler, R. (1)H NMR metabolomic study of auxotrophic starvation in yeast using Multivariate Curve Resolution-Alternating Least Squares for Pathway Analysis. *Sci Rep* **6**, 30982 (2016).
27. Imura, M., Iwakiri, R., Bamba, T. & Fukusaki, E. Metabolomics approach to reduce the Crabtree effect in continuous culture of *Saccharomyces cerevisiae*. *J Biosci Bioeng* **126**, 183-188 (2018).
28. Fabregat, A. et al. The Reactome Pathway Knowledgebase. *Nucleic Acids Research* **46**, 649-655 (2018).
29. Caspi, R. et al. The MetaCyc database of metabolic pathways and enzymes and the BioCyc collection of pathway/genome databases. *Nucleic Acids Research* **44**, 471-480 (2016).
30. Correia, K. & Mahadevan, R. Pan-genome-scale network reconstruction: a framework to increase the quantity and quality of metabolic network. (2018).
31. Thiele, I. & Palsson, B.O. A protocol for generating a high-quality genome-scale metabolic reconstruction. *Nat Protoc* **5**, 93-121 (2010).
32. Dias, O., Pereira, R., Gombert, A.K., Ferreira, E.C. & Rocha, I. iOD907, the first genome-scale metabolic model for the milk yeast *Kluyveromyces lactis*. *Biotechnol J* **9**, 776-790 (2014).
33. Onofre, S.B., Bertoldo, I.C., Abatti, D. & Refosco, D. Chemical Composition of the Biomass of *Saccharomyces cerevisiae* - (Meyen ex E. C. Hansen, 1883) Yeast obtained from the Beer Manufacturing Process. *International Journal of Environment, Agriculture and Biotechnology* **2**, 558-562 (2017).
34. Rimareva, L.V. et al. Study of intracellular ion composition of yeast *Saccharomyces cerevisiae* biomass. *Russian Agricultural Sciences* **43**, 186-189 (2017).
35. Hucker, B., Wakeling, L. & Vriesekoop, F. Vitamins in brewing: presence and influence of thiamine and riboflavin on wort fermentation. **122**, 126-137 (2016).
36. Kozak, B.U. et al. Replacement of the *Saccharomyces cerevisiae* acetyl-CoA synthetases by alternative pathways for cytosolic acetyl-CoA synthesis. *Metab Eng* **21**, 46-59 (2014).
37. Pallotta, M.L. Evidence for the presence of a FAD pyrophosphatase and a FMN phosphohydrolase in yeast mitochondria: a possible role in flavin homeostasis. *Yeast* **28**, 693-705 (2011).
38. Patring, J.D., Jastrebova, J.A., Hjortmo, S.B., Andlid, T.A. & Jagerstad, I.M. Development of a simplified method for the determination of folates in baker's yeast by HPLC with ultraviolet and fluorescence detection. *Journal of agricultural and food chemistry* **53**, 2406-2411 (2005).
39. Patring, J.D.M., Jastrebova, J.A., Hjortmo, S.B., Andlid, T.A. & Jägerstad, I.M. Development of a Simplified Method for the Determination of Folates in Baker's Yeast by HPLC with Ultraviolet and Fluorescence Detection. *Journal of agricultural and food chemistry* **53**, 2406-2411 (2005).
40. Pallotta, M.L. Evidence for the presence of a FAD pyrophosphatase and a FMN phosphohydrolase in yeast mitochondria: a possible role in flavin homeostasis. **28**, 693-705 (2011).
41. Buchfink, B., Xie, C. & Huson, D.H. Fast and sensitive protein alignment using DIAMOND. *Nature Methods* **12**, 59-60 (2014).
42. King, Z.A. et al. BiGG Models: A platform for integrating, standardizing and sharing genome-scale models. *Nucleic Acids Research* **44**, 515-522 (2016).
43. Kanehisa, M. & Goto, S. KEGG: kyoto encyclopedia of genes and genomes. *Nucleic acids research* **28**, 27-30 (2000).
44. Huerta-Cepas, J. et al. eggNOG 4.5: a hierarchical orthology framework with improved functional annotations for eukaryotic, prokaryotic and viral sequences. *Nucleic Acids Research* **44**, 286-293 (2016).
45. Peter, J. et al. Genome evolution across 1,011 *Saccharomyces cerevisiae* isolates. *Nature* **556**, 339-344 (2018).
