# Supplementary material for: A consensus S. cerevisiae metabolic model Yeast8 and its ecosystem for comprehensively probing cellular metabolism
Source: Nat Commun. 2019 Aug 8;10:3586. doi: 10.1038/s41467-019-11581-3 (PMC6687777; doi:10.1038/s41467-019-11581-3)
Supplement: Supplementary file 3 — Description of Additional Supplementary Files [file 41467_2019_11581_MOESM3_ESM.pdf]

Description of Additional Supplementary Files

File Name: Supplementary Data 1
Description: Biolog growth test results for S. cerevisiae S288c under different substrate sources (green color means that the related substrates could be used by this strain)
