# Supplementary material for: A consensus S. cerevisiae metabolic model Yeast8 and its ecosystem for comprehensively probing cellular metabolism
Source: Nat Commun. 2019 Aug 8;10:3586. doi: 10.1038/s41467-019-11581-3 (PMC6687777; doi:10.1038/s41467-019-11581-3)
Supplement: Supplementary file 5 — Reporting Summary [file 41467_2019_11581_MOESM5_ESM.pdf]

nature research

Corresponding author(s): Jens Nielsen

Last updated by author(s): Apr 6, 2019

# Reporting Summary

Nature Research wishes to improve the reproducibility of the work that we publish. This form provides structure for consistency and transparency in reporting. For further information on Nature Research policies, see Authors & Referees and the Editorial Policy Checklist.

## Statistics

For all statistical analyses, confirm that the following items are present in the figure legend, table legend, main text, or Methods section.

| n/a | Confirmed | |
|---|---|---|
| ☐ | ☒ | The exact sample size ($n$) for each experimental group/condition, given as a discrete number and unit of measurement |
| ☒ | ☐ | A statement on whether measurements were taken from distinct samples or whether the same sample was measured repeatedly |
| ☐ | ☒ | The statistical test(s) used AND whether they are one- or two-sided<br>*Only common tests should be described solely by name; describe more complex techniques in the Methods section.* |
| ☒ | ☐ | A description of all covariates tested |
| ☒ | ☐ | A description of any assumptions or corrections, such as tests of normality and adjustment for multiple comparisons |
| ☒ | ☐ | A full description of the statistical parameters including central tendency (e.g. means) or other basic estimates (e.g. regression coefficient) AND variation (e.g. standard deviation) or associated estimates of uncertainty (e.g. confidence intervals) |
| ☐ | ☒ | For null hypothesis testing, the test statistic (e.g. $F$, $t$, $r$) with confidence intervals, effect sizes, degrees of freedom and $P$ value noted<br>*Give P values as exact values whenever suitable.* |
| ☒ | ☐ | For Bayesian analysis, information on the choice of priors and Markov chain Monte Carlo settings |
| ☒ | ☐ | For hierarchical and complex designs, identification of the appropriate level for tests and full reporting of outcomes |
| ☒ | ☐ | Estimates of effect sizes (e.g. Cohen's $d$, Pearson's $r$), indicating how they were calculated |

*Our web collection on statistics for biologists contains articles on many of the points above.*

## Software and code

Policy information about availability of computer code

| | |
|---|---|
| Data collection | Webdata on protein 3D structure's parameters were scraped using Python (2.7.16), Python (3.6.1), the beautifulsoup4 library (4.7.1), the biopython library (1.7.3), the pypdb library (1.103). |
| Data analysis | The yeast GEM curation and update is conducted using Cobra toolbox (3.0.0) and RAVEN toolbox (2.0.0) based on Matlab (R2017b). Linear programming problems for FBA and FVA were constructed using Cobra toolbox (3.0.0) and RAVEN toolbox (2.0.0) and solved using GLPK (4.65) and Gurobi (8.1.0). ecYeast was produced using GECKO toolbox (1.3.4) based on Matlab (R2017b). panYeast8, coreYeast8 and strain specific model were constructed in Matlab (2017b) using Cobra toolbox (3.0.0) and RAVEN toolbox (2.0.0). CLUMPS, HotSpot and statistical analysis were conducted in the R environment (3.5.2). |

For manuscripts utilizing custom algorithms or software that are central to the research but not yet described in published literature, software must be made available to editors/reviewers. We strongly encourage code deposition in a community repository (e.g. GitHub). See the Nature Research guidelines for submitting code & software for further information.

## Data

Policy information about availability of data

All manuscripts must include a data availability statement. This statement should provide the following information, where applicable:

- Accession codes, unique identifiers, or web links for publicly available datasets
- A list of figures that have associated raw data
- A description of any restrictions on data availability

The genome information for 1011 yeast strains could be found in http://1002genomes.u-strasbg.fr/.
The other associated data used in this work could be found in the followed four repositories on GitHub:
https://github.com/SysBioChalmers/yeast-GEM
https://github.com/SysBioChalmers/ecModels (containing raw data for the Figure.2)
https://github.com/SysBioChalmers/panYeast-GEM (containing raw data for the Figure.3)

https://github.com/SysBioChalmers/proYeast-GEM (containing raw data for Figure 4 and Figure 5)

# Field-specific reporting

Please select the one below that is the best fit for your research. If you are not sure, read the appropriate sections before making your selection.

☒ Life sciences ☐ Behavioural & social sciences ☐ Ecological, evolutionary & environmental sciences

For a reference copy of the document with all sections, see nature.com/documents/nr-reporting-summary-flat.pdf

# Life sciences study design

All studies must disclose on these points even when the disclosure is negative.

| | |
|---|---|
| Sample size | Sample sizes were chose as described in the Methods section according to the need in the related analysis. |
| Data exclusions | When conducting the mutation mapping analysis based on protein 3D structures, the protein 3D structures of low quality were removed before analysis. This procedure is described in the Methods section. |
| Replication | In 96-well plates cultivation, three parallel experiments were conducted for each condition. |
| Randomization | No new experiments were conducted for this computational study. |
| Blinding | No new experiments were conducted for this computational study. |

# Reporting for specific materials, systems and methods

We require information from authors about some types of materials, experimental systems and methods used in many studies. Here, indicate whether each material, system or method listed is relevant to your study. If you are not sure if a list item applies to your research, read the appropriate section before selecting a response.

## Materials & experimental systems

| n/a | Involved in the study | |
|---|---|---|
| ☒ | ☐ | Antibodies |
| ☐ | ☒ | Eukaryotic cell lines |
| ☒ | ☐ | Palaeontology |
| ☒ | ☐ | Animals and other organisms |
| ☒ | ☐ | Human research participants |
| ☒ | ☐ | Clinical data |

## Methods

| n/a | Involved in the study | |
|---|---|---|
| ☒ | ☐ | ChIP-seq |
| ☒ | ☐ | Flow cytometry |
| ☒ | ☐ | MRI-based neuroimaging |

## Eukaryotic cell lines

Policy information about cell lines

| | |
|---|---|
| Cell line source(s) | Saccharomyces cerevisiae S288C |
| Authentication | NO |
| Mycoplasma contamination | NO |
| Commonly misidentified lines (See ICLAC register) | NO |
